# Assessment of Clinical Characteristics and Outcomes of Liver Diseases Unique to Pregnancy at a Tertiary Hospital in Ethiopia: A Retrospective Cohort Study

**DOI:** 10.1155/2022/9894407

**Published:** 2022-12-19

**Authors:** Sintayehu Mekonnen, Henok Fisseha, Tewodros Getinet

**Affiliations:** ^1^Department of Internal Medicine, All Africa Leprosy, Tuberculosis and Rehabilitation Training Center, Addis Ababa, Ethiopia; ^2^Department of Internal Medicine, St. Paul's Hospital Millennium Medical College, Addis Ababa, Ethiopia; ^3^School of Public Health, St. Paul's Hospital Millennium Medical College, Addis Ababa, Ethiopia

## Abstract

**Background:**

Liver disease is a rare complication of pregnancy that can lead to several consequences and require specific intervention with implications for both the mother and fetus. This study is aimed at assessing the clinical profile and associated complications of liver diseases unique to pregnancy at St. Paul's Hospital Millennium Medical College, Addis Ababa, Ethiopia. *Methodology*. This study is a retrospective cohort study of all identified cases admitted to the obstetrics ward and intensive care unit (ICU) from January 2018 to December 2020 at St. Paul's Hospital Millennium Medical College, Addis Ababa, Ethiopia. Medical records were reviewed for clinical features, biochemical profiles, and fetomaternal complications. Data were analyzed using SPSS version 26. A chi-square test was done to look for an association with a *p* value less than 0.05 considered statistically significant, and an odds ratio was determined to assess the effect size.

**Results:**

From 95 cases identified, preeclampsia/eclampsia with liver dysfunction accounted for 43 (45%), followed by hemolysis elevated liver enzyme and low platelet (HELLP syndrome) 35 (36.8%), hyperemesis gravidarum with liver dysfunction 9 (9.5%), acute fatty liver of pregnancy (AFLP) 7 (7.4%), and intrahepatic cholestasis of pregnancy 1 (1.1%). When compared to HELLP syndrome, AFLP showed significantly higher median (IQR) values (*p* < 0.05) for total bilirubin 13.3 (7.3-16.3), direct bilirubin 9.73 (6.87-11.9) mg/dL, prothrombin time 23 (20.4-25.7) seconds, international normalization ratio 2.2 (1.9-2.4), white blood count 23.8 (17.8-26.6)^∗^10^3^/*μ*L, creatinine 3.5 (2.44-5.6) mg/dL, and lower hemoglobin level of 7.9 (6.2-10) g/dL. There were 4 (4.2%) maternal hospital deaths, with a case fatality rate of HELLP syndrome being 8.6% and 14.3% in AFLP. The overall hospital fetal mortality was 33 (34.7%). In this study, 42 patients with HELLP syndrome and AFLP had an increased risk of maternal ICU admission (OR = 25.5, 95% CI: 5.48-118.6, *p* value = 0.001), acute kidney injury requiring dialysis (OR = 12.2, 95% CI: 1.46-102.2, *p* value = 0.009), placental abruption (OR = 14.2, 95% CI: 1.72-117.1, *p* value = 0.004), and stillbirth (OR = 7.2, 95% CI: 2.38-21.7, *p* value = 0.001).

**Conclusion:**

Preeclampsia with liver dysfunction and HELLP syndrome accounted for the majority of cases. It also demonstrated key biochemical characteristics that can be used to distinguish between HELLP syndrome and AFLP. Emphasis has to be given to the risk of requiring maternal ICU admission, dialysis, abruption of the placenta, and stillbirths while managing patients diagnosed with HELLP syndrome and AFLP.

## 1. Introduction

Liver disease is an uncommon complication of pregnancy with a 3% prevalence [1] that poses a clinical challenge for hepatologists and obstetricians. Some pregnancy-unique liver diseases can have deadly outcomes for both the mother and the fetus if they are not detected and treated in time [2–5].

Liver disease unique to pregnancy is classified into five categories that include intrahepatic cholestasis of pregnancy (ICP), hyperemesis gravidarum (HG), acute fatty liver of pregnancy (AFLP), hemolysis elevated liver enzyme and low platelet count (HELLP) syndrome, and preeclampsia (PE)/eclampsia (E) with liver dysfunction. Even though their particular trimester occurrence and clinical symptoms can help with diagnosis, it is frequently challenging to distinguish between AFLP, HELLP syndrome, and PE/E with liver dysfunction because of the overlap in their clinical profiles [6, 7]. ICP has also been associated with the development of AFLP [8] and preeclampsia [9].

In addition to the clinical difficulty, pregnancy-specific liver illness can lead to several complications, including the need for cesarean delivery, low birth weight, acute renal injury requiring dialysis, abruption of the placenta, liver hematoma, maternal and neonatal ICU admissions, and even fatal outcomes. In comparison to developed countries, where there were zero maternal and 1-4% fetal hospital mortality reports [1, 7], developing countries had higher rates of maternal hospital mortality of 20–25% and fetal hospital mortality of 32.5–35% [10, 11]. Therefore, rapid diagnosis and measures to address complications are necessary for the prevention of fatal outcomes [6, 12].

To our knowledge, there are no published studies on liver disease unique to pregnancy in Ethiopia besides a case report of acute fatty liver of pregnancy [13] and studies on HELLP syndrome under the heading hypertensive disease of pregnancy [14–16]. In a country where maternal health is prioritized as a top public health concern, this can be considered a shortcoming.

In light of the scarcity of literature, this study is aimed at describing the demographics, clinical characteristics, biochemical features, and consequences of pregnancy-specific liver disease. Additionally, it examines the associations between the consequences of HELLP syndrome and AFLP in relation to other etiologic causes of liver disease unique to pregnancy. This study will serve as a guide for clinical judgment and set the path for future studies.

## 2. Methods

### 2.1. Study Area, Period, and Design

This retrospective cohort study on liver disease unique to pregnancy was conducted at St. Paul's Hospital Millennium Medical College (SPHMMC), a referral and teaching hospital found in Addis Ababa, Ethiopia [17].

St. Paul's Hospital Millennium Medical College is the second-largest hospital in the country established through a decree of the Council of Ministers in 2010, although the medical school opened in 2007 and the late Emperor Haile Selassie established the hospital in 1968. It is under the Ethiopian Federal Health Ministry management with >8000 deliveries per year receiving patients from 10 catchment health centers and numerous other hospitals in Ethiopia. On average, daily, the hospital gives medical services to 1,200 patients with diversified complaints in several specialty and subspecialty fields. The gastroenterology and hepatology unit runs under the department of internal medicine with five subspecialists and has been providing outpatient and inpatient services aided by endoscopy and colonoscopy evaluation of patients since 2011GC.

The study population was all pregnant mothers above 18 years of age diagnosed with liver diseases unique to a pregnancy and admitted to the obstetrics ward and ICU of SPHMMC from January 1, 2018, to December 31, 2020. The source population was all pregnant mothers, aged above 18, admitted to SPHMMC at the obstetrics ward and ICU during the study period.

The study included patients diagnosed with liver diseases unique to pregnancy with serum aspartate aminotransferase (AST) > 40 U/L/alanine aminotransferase (ALT) > 40 U/L or total bilirubin > 1 mg/dL. Subjects with known liver diseases such as acute and chronic hepatitises A, B, C, D, and E before and at admission, alcoholic or nonalcoholic fatty liver disease, the Budd-Chiari syndrome, primary biliary cirrhosis, primary sclerosing cholangitis, and gallbladder diseases were excluded.

### 2.2. Operational Definition

(1) Acute fatty liver of pregnancy (AFLP): diagnosed using the Swansea criteria, where six or more of the following features are in absence of another explanation [1, 18]:
  - Vomiting
  - Abdominal pain
  - Polydipsia/polyuria
  - Encephalopathy
  - Elevated bilirubin (>0.8 mg/dL or>14 *μ*mol/L)
  - Hypoglycemia (glucose < 72 mg/dL or < 4 mmol/L)
  - Leukocytosis (>11,000 cells/*μ*L)
  - Elevated transaminases (AST or ALT) (>42 international unit/L)
  - Elevated ammonia (>47 *μ*mol/L)
  - Elevated urate (5.7 mg/dL or >340 *μ*mol/L)
  - Acute kidney injury or creatinine > 1.7 mg/dL (150 *μ*mol/L)
  - Coagulopathy or prothrombin time > 14 seconds
  - Ascites or bright liver on ultrasound scan
  - Microvesicular steatosis on liver biopsy
(2) HELLP syndrome: patients diagnosed using the Mississippi criteria [19]:
  - Platelet count ≤ 150,000 cells/*μ*L
  - Plus LDH > 600 IU/L
  - Plus AST or ALT ≥ 40 IU/L
(3) Intrahepatic cholestasis of pregnancy (ICP) [7]:
  - Presence of pruritus and elevated aminotransferases > 40 U/L
(4) Preeclamptic liver dysfunction [7]:
  - Elevated aminotransferases > 40 U/L or bilirubin > 1 mg/dL in the presence of hypertension, proteinuria, and/or edema after 20 weeks of gestation
(5) Hyperemesis gravidarum with liver dysfunction [7]:
  - Elevated aminotransferases > 40 IU/L or bilirubin > 1 mg/dL in the presence of persistent vomiting for more than one week during the first or second trimester
(6) Acute kidney injury:
  - Serum creatinine above 1.2 mg/dL
(7) Acute kidney injury (AKI) requiring hemodialysis (HD):
  - AKI of any degree where the patient needs HD support
(8) Laboratory values:
  - Data that is the maximum value when the specific diagnosis was made
(9) Overall fetal hospital mortality [7]:
  - The sum of miscarriage, stillbirth, and early neonatal death

### 2.3. Data Collection Method and Tools

**Figure 1:**
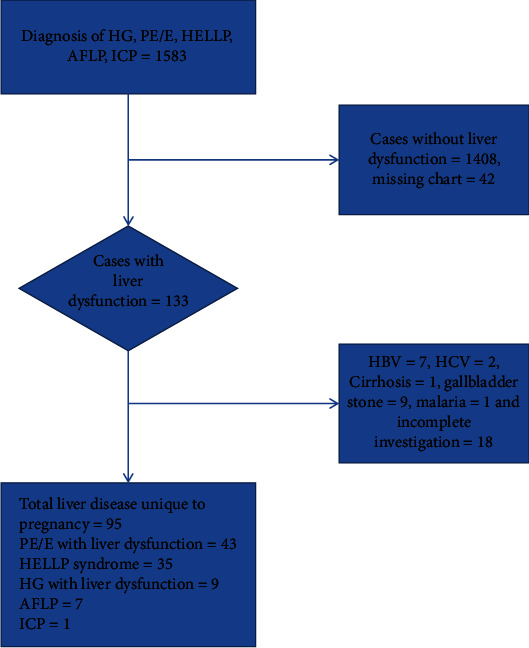
Selection flow chart of liver disease unique to pregnancy at St. Paul's Hospital Millennium Medical College. AFLP: acute fatty liver of pregnancy; HELLP: hemolysis elevated liver enzymes and a low platelet; PE/E: preeclampsia/eclampsia; HG: hyperemesis gravidarum; ICP: intrahepatic cholestasis; HBV: hepatitis B virus; HCV: hepatitis C virus.

From the hospital health management information system (HMIS) registry book, all cases with admission and discharge diagnoses of ICP, HG, preeclampsia, eclampsia, HELLP syndrome, and/or AFLP were first collected. Only those who met the inclusion criteria underwent additional testing (Figure 1). Crosschecking the handover books of the ward nurses allowed for the identification of cases that might have gone unnoticed due to improperly recorded patient diagnoses in the HMIS. The data from medical charts were extracted using a structured data abstraction tool, which is composed of diagnosis, demographic characteristics, clinical profile, biochemical workup, and outcomes. Maternal and fetal outcomes include cesarean delivery, abruption placenta, maternal ICU admission, acute kidney injury requiring dialysis, liver capsular hematoma/rupture, sex of the fetus, birth weight, neonatal ICU admission, miscarriage, stillbirth, early neonatal, and maternal death.

### 2.4. Statistical Analysis

The Statistical Package for the Social Sciences (SPSS) version 26.0 was used to analyze the data. Descriptive statistics such as median, interquartile range (IQR), percentages, and ratios were used to describe the demographic, clinical characteristics, biochemical profile, and outcomes of liver disease during pregnancy. Data distribution for the continuous variable was checked using the Shapiro-Wilk test, and all biochemical tests showed nonparametric distribution. The Mann–Whitney test was done to compare HELLP syndrome and AFLP. Cross tabulation was used to compare different categorical variables and subjected to chi-square analysis with statistical association considered significant when *p* value is less than 0.05. Fisher's exact test was used when assumptions were violated. The odds ratio with confidence interval was examined to look for effect size.

Frequency was generated to look for missing data. The analysis excluded variables including polyuria, polydipsia, uric acid, random blood sugar, and bile acid that had a large amount of missing data. The variables with some missing data, such as total bilirubin, direct bilirubin, prothrombin time, and INR, were subjected to missing value analysis; nevertheless, the probability of missing data was the same in all cases, with Little's MCAR (missing completely at random) test *p* value being 0.085. The data was analyzed based on available data for each variable or pairwise deletion.

Before conducting the research, permission was obtained from SPHMMC institutional review board.

## 3. Results

Preeclampsia/eclampsia with liver dysfunction accounted for 43 (45%) of the 95 cases of liver disease specific to pregnancy during the study period, followed by hemolysis with elevated liver enzyme and low platelet levels, 35 (36.8%); acute fatty liver of pregnancy, 7 (7.3%); hyperemesis gravidarum with liver dysfunction, 9 (9.5%); and intrahepatic cholestasis of pregnancy, 1 (1.1%).

### 3.1. Demographic and Obstetrics Characteristics

The median (IQR) age of mothers was 26 (22-30), ranging from 18 to 36 years. More than half were primiparous 53.68%, multiple pregnancies occurred in 9.5%, 84.2% of patients were diagnosed in the third trimester, and 4.2% were diagnosed postpartum. More than half of the newborns had a low birth weight 68.4% (Table 1).

### 3.2. Clinical Manifestation of Liver Disease

Headache, peripheral edema, and proteinuria were the most common symptoms in patients with preeclampsia/eclampsia with liver dysfunction in 39.5%, 37.2%, and 88.4% of cases, respectively. In HELLP syndrome, proteinuria was present in 82.9% of cases, along with abdominal pain in 68.6% of cases and seizures in 45.7% of cases. All AFLP patients experienced nausea, vomiting, and abdominal pain, and 42.9% of them had proteinuria. All HG patients with liver dysfunction presented with nausea/vomiting, and 33.3% also had abdominal pain. The only clinical symptom of ICP was pruritus (Table 2).

### 3.3. Biochemical Characteristics

Comparing the laboratory profiles of patients with AFLP and those with HELLP syndrome revealed significantly higher median (IQR) values for total bilirubin 13.3 (7.3-16.3) mg/dL/2.9 (1.2-5.5) mg/dL, direct bilirubin 9.73 (6.87-11.9) mg/dL/1.39 (0.56-4.37) mg/dL, prothrombin time 23 (20.4-25.7) seconds/13.9 (11.9-20.3) seconds, international normalization ratio 2.2 (1.9-2.4)/1.1 (0.9-1.7), white blood count 23.8 (17.8-26.6)^∗^10^3^/*μ*L/12.8 (10.6-18.9) 10^3^/*μ*L, and creatinine 3.5 (2.44-5.6) mg/dL/1.45(0.71-3.4) mg/dL, respectively (*p* < 0.05). However, AFLP syndrome had hemoglobin levels that were much lower than HELLP syndrome: 7.9 (6.2-10) g/dL/12.2 (9.1-14) g/dL (*p* < 0.05) (Table 3).

### 3.4. Maternal and Fetal Outcomes of Liver Disease Unique to Pregnancy

Among maternal outcomes, there were 40 (42.1%) cesarean section deliveries, nine dialyses for AKI (9.5%), ten abruptions of the placenta (10.5%), one liver hematoma, and 23 maternal ICU admissions (24.2%). There were four maternal hospital mortalities (4.2%), one occurred in AFLP and three from HELLP syndrome (Table 4).

Regarding fetal outcomes, there were 16 (16.8%) neonatal ICU admissions, six miscarriages of pregnancy (6.3%), 23 (24.2%) stillbirths, and four early neonatal deaths (4.2%), making the overall fetal hospital mortality 33 (34.7%) (Table 4).

Patients with HELLP syndrome and AFLP were more likely to be admitted to the maternal intensive care unit (OR = 25.5, 95% CI: 5.48-118.6, *p* = 0.001), suffer an acute kidney injury that required dialysis (OR = 12.2, 95% CI: 1.46-102.2, *p* = 0.009), experience placental abruption (OR = 14.2, 95% CI: 1.72-117.1, *p* = 0.004), and have a stillbirth (OR = 7.2, 95% CI: 2.38-21.7, *p* = 0.001) (Table 4).

## 4. Discussion

Liver disease specific to pregnancy is an uncommon condition and is often associated with diagnostic challenges and fatal consequences. In this study, we evaluated 95 cases and preeclampsia/eclampsia with liver dysfunction and HELLP syndrome take the highest proportions like previous studies [1, 7, 11], but on the contrary, ICP was found to be uncommon and the least etiologic causes of liver disease unique to pregnancy.

Despite the lack of data on ICP in Ethiopia to the authors' knowledge, studies from the United States of America indicate that it is uncommon in African Americans and the prevalence varies by ethnicity and geographic region [7]. Since SPHMMC is a tertiary care hospital that accepts complex and referred cases, this study might have underestimated the prevalence. In addition, inpatient admission and referral may not be necessary in the majority of ICP cases. The uncommon diagnosis of ICP may also be attributed to the hospital's absence of serum bile testing.

Multiparity was seen as a risk factor for preeclampsia/eclampsia with liver dysfunction and HELLP syndrome, whereas multiparity and multiple gestations are considered risk factors for AFLP [6]. In this study, 56.1% of the women were multiparous and all had singleton births, whereas in a Chinese study, 30% of the women were multiparous and 91% had singleton birth [20]. According to prior studies, AFLP is frequently found in male fetuses, and in this study, 6 of the newborns (85.7%) were male [7, 20, 21]. Similar to the earlier Ethiopian study, 62.9% of women with HELLP syndrome and 44.2% of those with preeclampsia/eclampsia with liver dysfunction were primiparous and no association was detected between HELLP syndrome, preeclampsia/eclampsia, and parity (*p* = 0.091, 0.171) [22].

It is often difficult to differentiate some liver diseases unique to pregnancy due to the clinical overlap and lack of fulfillment of all the diagnostic criteria. In this study, some patients with preeclampsia/eclampsia with liver dysfunction had elevated transaminases with low platelet which does not meet the criteria for HELLP syndrome [23]. A study from the United Kingdom [1] that highlights HELLP syndrome as a spectrum of preeclamptic liver dysfunction also made the same observation. Proteinuria, which is regarded to be a sign of HELLP syndrome and preeclamptic liver dysfunction, was discovered in 42.9% of AFLP, which is similar to a prior study [24]. As a result, it is critical to have a strong clinical suspicion of AFLP in patients who have proteinuria and liver dysfunction that cannot be fully explained by those conditions.

In a previous case report from Ethiopia, the patient's initial suspected diagnosis of HELLP syndrome was later modified to AFLP following a clinical reevaluation [13]. To provide diagnostic clues, previous studies showed that the most notable biochemical characteristic of AFLP was a greater level of bilirubin, an elevated prothrombin time, an increased international normalized ratio, and a mild increase in aminotransferases when compared to HELLP syndrome [24]. Another study recommended taking the diagnosis of AFLP into account when there was a considerable increase in aminotransferase > 200 and direct bilirubin level > 3.5 [20]. This study found that patients with AFLP had significantly a higher median value in serum total bilirubin, direct bilirubin, prothrombin time, international normalization ratio, white blood count, and creatinine while a significantly lower hemoglobin level than HELLP syndrome with *p* value less than 0.05. This finding can help physicians with the clinical differentiation of these diseases. Although the birth of the fetus is the mainstay of management in both disorders, the urgency of intervention and disease-specific fetal complications necessitate expert involvement for quick recognition and treatment when difficulties arise.

Fetomaternal consequences including abruption of the placenta (*p* = 0.004), maternal ICU admission (*p* = 0.001), acute kidney injury requiring dialysis (*p* = 0.009), and stillbirth (*p* = 0.001) were particularly common in individuals with AFLP and HELLP syndrome. This finding helps physicians in resource-constrained countries like ours to anticipate and make early decisions about where to treat patients with these conditions. It also provides information for policymakers to expand setups that can offer ICU care and dialysis services to make comprehensive treatment available throughout the regions.

Although maternal and fetal hospital mortality in this study is comparable to the Indian study [11], it is greater than the reports from developed countries [1, 7]. This may be because the study area is a tertiary referral hospital, where patients with this illness usually present after developing some complications and may not be adequately diagnosed early enough for intervention. Additionally, it has been shown that maternal clinical diagnosis, severity of the clinical illness, low birth weight, and preterm birth are independent contributing factors for stillbirths in Ethiopia [25]. More than half of the newborns in this study had complications due to low birth weight, which could be another contributing factor. This study also found one liver hematoma, which was previously reported to be an uncommon consequence of the severe form of HELLP syndrome [24]. This finding emphasizes the patient's delayed presentation to a treatment facility.

In terms of maternal hospital mortalities, sepsis caused one death in a patient with AFLP who delivered birth at 34 weeks. The remaining occurred in HELLP syndrome, in which the causes of maternal mortality were disseminated intravascular coagulation, cerebral hemorrhage, and multiorgan failure because of ICU-related complications. The pregnancies ended at 28, 32, and 37 weeks, respectively. The last patient underwent a perimortem cesarean section for resuscitation purposes on her immediate arrival day after the diagnosis of HELLP syndrome was made, but she died after a week-long stay in ICU following cardiopulmonary resuscitation. This, once more, highlights the importance of early diagnosis and management of these conditions to avoid fatal outcomes. According to a previous study, the prevention of infection and bleeding complications that could arise from these disorders will probably lessen fatal results [24].

Outcomes in hyperemesis gravidarum with liver dysfunction and ICP were favorable, and no maternal or newborn morbidity or mortality was seen, similar to previous studies [1, 7]. However, there were reports of stillbirths and low birth weight in ICP [26]. This may necessitate a more extensive study involving more cases.

To the best of our knowledge, this is the first study carried out in Ethiopia that provides a thorough clinical picture as well as an analysis of the burden of liver disease related to pregnancy. It has several limitations, though, including the possibility of referral bias as it was done in a single tertiary referral institution. Due to the retrospective study's design, secondary clinical data had to be used, which led to incomplete investigations, missing charts, and inadequate documentation. The observed statistical association in this study needs to be reproduced with a bigger sample size because the confidence interval was broad and multivariate analysis was not done due to the small number of maternal and fetal outcomes.

## 5. Conclusion

In this study, preeclampsia with liver dysfunction and HELLP syndrome are the most common causes of liver diseases unique to pregnancy similar to reports across the globe; however, ICP was found to be uncommon. It also demonstrated key biochemical characteristics that can be used to distinguish between HELLP syndrome and AFLP. Patients diagnosed with acute fatty liver of pregnancy and HELLP syndrome are at risk for maternal ICU admission, requiring dialysis, developing abruption of the placenta, and stillbirths. This highlights the importance of early diagnosis, close monitoring, and timely referral to a higher center when necessary. It also emphasizes the requirement for more health facilities to be upgraded and equipped to provide ICU and dialysis services to reduce fetal and maternal fatalities. Further prospective studies with multivariate analysis are needed to determine factors associated with maternal and fetal hospital mortality.

## Figures and Tables

**Table 1:** Patient demographic and obstetrics characteristics.

		PE/E with liver dysfunction *N* (%)	HELLP syndrome *N* (%)	AFLP *N* (%)	ICP *N* (%)	HG with liver dysfunction *N* (%)
Age	≤24	13 (30.2%)	12 (34.3%)	4 (57.1%)	—	8 (88.9%)
	25-34	25 (58.1%)	18 (51.4%)	3 (42.9%)	1 (100%)	1 (11.1%)
	≥35	5 (11.6%)	5 (14.3%)	—	—	—
Parity	Primipara	19 (44.2%)	22 (62.9%)	3 (42.9%)	1 (100%)	6 (66.7%)
	Multipara	24 (55.8%)	13 (37.1%)	4 (57.1%)	—	3 (33.3%)
Number of gestation	Single	38 (88.4%)	32 (91.4%)	7 (100%)	1 (100%)	8 (88.9%)
	Multiple	5 (11.6%)	3 (8.6%)	—	—	1 (11.1%)
Time of diagnosis	1st trimester	—	—	—	—	7 (77.8%)
	2nd trimester	4 (9.3%)	2 (5.7%)	—	—	2 (22.2%)
	3rd trimester	39 (90.7%)	29 (82.9%)	7 (100%)	1 (100%)	—
	Postpartum	—	4 (11.4%)	—	—	—
Birth weight	<1500	16 (37.2%)	10 (28.6%)	2 (28.6%)	—	—
	1500-2499	15 (34.9%)	18 (51.4%)	3 (42.9%)	—	1 (11.1%)
	>2500	12 (27.9%)	7 (20%)	2 (28.6%)	1 (100%)	8 (88.9%)
Sex of newborn	Male	21 (48.8%)	21 (60%)	6 (85.7%)	1 (100%)	5 (55.6%)
	Female	22 (51.2%)	14 (40%)	1 (14.3%)	—	4 (44.4%)

PE/E: preeclampsia/eclampsia; HELLP: hemolysis elevated liver enzyme and low platelet; AFLP: acute fatty liver of pregnancy; ICP: intrahepatic cholestasis of pregnancy; HG: hyperemesis gravidarum.

**Table 2:** Clinical manifestations of liver disease specific to pregnancy.

	PE/E with liver dysfunction *N* (%)	HELLP syndrome *N* (%)	AFLP *N* (%)	HG with liver dysfunction *N* (%)
Nausea and vomiting	4 (9.3%)	9 (25.7%)	7 (100%)	9 (100%)
Headache	17 (39.5%)	13 (37.1%)	1 (14.3%)	—
Abdominal pain	10 (23.3%)	24 (68.6%)	7 (100%)	3 (33.3%)
Oliguria	2 (4.7%)	5 (14.3%)	4 (57.1%)	—
Edema	16 (37.2%)	10 (28.6%)	4 (57.1%)	—
Encephalopathy	2 (4.7%)	12 (34.3%)	4 (57.1%)	—
Ascites	—	8 (22.9%)	5 (71.4%)	—
Jaundice	—	11 (31.4%)	6 (85.7%)	—
Convulsion	6 (14%)	16 (45.7%)	1 (14.3%)	—
Proteinuria	38 (88.4%)	29(82.9%)	3 (42.9%)	—

PE/E: preeclampsia/eclampsia; HELLP: hemolysis elevated liver enzyme and low platelet; AFLP: acute fatty liver of pregnancy; HG: hyperemesis gravidarum.

**Table 3:** Biochemical characteristics of liver disease specific to pregnancy.

	PE/E with liver dysfunction	HELLP syndrome	AFLP	HG with liver dysfunction	*p* value for AFLP and HELLP
Aspartate amino transferase (UI/L)	105.9 (83-180)	276 (120-700)	322 (140-507)	124.5 (96-147.2)	0.921
Alanine aminotransferase (UI/L)	75 (48.8-159.3)	225.8 (115.7-379.6)	87 (43-231)	81.8 (54.6-109.6)	0.076
Total bilirubin (mg/dL)	0.4 (0.2-1.1)	2.9 (1.2-5.5)	13.3 (7.3-16.3)	0.7 (0.5-0.8)	0.005
Direct bilirubin (mg/dL)	0.21 (0.11-0.45)	1.39 (0.56-4.37)	9.73 (6.87-11.9)	0.18 (0.14-0.2)	0.002
Prothrombin time (s)	13 (12-14.6)	13.9 (11.9-20.3)	23 (20.4-25.7)	11.5 (9.7-13)	0.007
International normalization ratio	1.1 (1-1.1)	1.1 (0.9-1.7)	2.2 (1.9-2.4)	0.9 (0.8-1)	0.006
Lactate dehydrogenase	411 (292-516)	1195 (888-2100)	944 (800-1950)	—	0.428
White blood count (cells^∗^10^3^/*μ*L)	10.6 (8-12.4)	12.8 (10.6-18.9)	23.8 (17.8-26.6)	8.9 (7.7-9.6)	0.002
Hemoglobin (g/dL)	13.7 (12.7-14.3)	12.2 (9.1-14)	7.9 (6.2-10)	12.1 (12-13.5)	0.026
Platelet (cells^∗^10^3^/*μ*L)	175 (157-195)	58 (44-83)	116 (42-183)	235 (207-280)	0.076
Creatinine (mg/dL)	0.7 (0.56-0.88)	1.45 (0.71-3.4)	3.5 (2.44-5.6)	0.67 (0.64-0.87)	0.032

Values are presented as a median and interquartile range. PE/E: preeclampsia/eclampsia; HELLP: hemolysis elevated liver enzyme and low platelet; AFLP: acute fatty liver of pregnancy; HG: hyperemesis gravidarum.

**Table 4:** Maternal and neonatal outcomes of liver disease specific to pregnancy.

	PE/E with liver dysfunction *N* (%)	HELLP syndrome *N* (%)	AFLP *N* (%)	HG with liver dysfunction *N* (%)	Total *N* (%)	*p* value (HELLP syndrome+AFLP)
Cesarean section	21 (48.8%)	16 (45.7%)	2 (28.6%)	1 (11.1%)	40 (42.1%)	0.895
AKI on dialysis	1 (2.3%)	6 (17.1%)	2 (28.6%)	—	9 (9.5%)	0.009
Abruption of placenta	1 (2.3%)	7 (20%)	2 (28.6%)	—	10 (10.5%)	0.004
Liver hematoma or rupture	—	1 (2.9%)	—	—	1 (1.1%)	0.442
ICU admission	2 (4.7%)	16 (45.7%)	5 (71.4%)	—	23 (24.2%)	0.001
Maternal death	—	3 (8.6%)	1 (14.3%)	—	4 (4.2%)	0.035
Miscarriage	4 (9.3%)	2 (5.7%)	—	—	6 (6.3%)	0.907
Neonatal ICU admission	9 (20.9%)	5 (14.3%)	2 (28.6%)	—	16 (16.8%)	0.849
Early neonatal death	2 (4.7%)	2 (5.7%)	—	—	4 (4.2%)	1.000
Stillbirth	5 (11.6%)	13 (37.1%)	5 (71.4%)	—	23 (24.2%)	0.001

PE/E: preeclampsia/eclampsia; HELLP: hemolysis elevated liver enzyme and low platelet; AFLP: acute fatty liver of pregnancy; HG: hyperemesis gravidarum; AKI: acute kidney injury; ICU: intensive critical care unit.

## Data Availability

The data used to support the findings of this study are available from the corresponding author upon request.
